# Usefulness of high-frequency ultrasonography in the diagnosis of odontogenic cutaneous fistula^☆^^☆☆^

**DOI:** 10.1016/j.abd.2020.06.013

**Published:** 2021-01-31

**Authors:** Arcadi Altemir-Vidal, Maribel Iglesias-Sancho, Monica Quintana-Codina

**Affiliations:** Dermatology Department, Hospital Universitari Sagrat Cor – Grupo Quironsalud, Barcelona, Spain

Dear Editor

A 27-year-old female patient presented with a six-month history of a persistent and painful nodule on the chin. She referred occasional purulent exudate. There was no history of fever or systemic symptoms. Several courses of oral antibiotics had been ineffective. In the extraoral examination she presented an erythematous nodule with an important surrounding retraction (Fig. 1). Ultrasonography (Esaote MyLab Gamma®, 18 MHz) revealed a hypoechoic lesion with increased blood flow in the base and periphery. Moreover, an inflamed tortuous sinus tract extended through the subcutaneous tissue to the alveolar bone (Fig. 2). These findings were consistent with an odontogenic cutaneous fistula (OCF). A panoramic radiograph showed its origin in a mandibular incisor (Fig. 3). The patient was referred to the Maxillofacial Surgery Department of this hospital for assessment and treatment. Dental extraction was suggested in order to remove the source of the infection.

**Figure 1:**
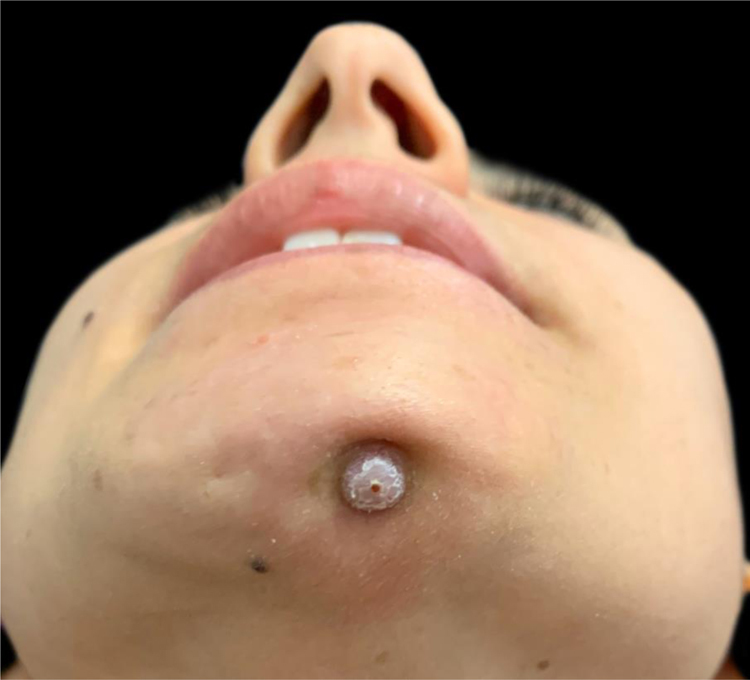
Erythematous nodule with surrounding retraction on the chin.

**Figure 2:**
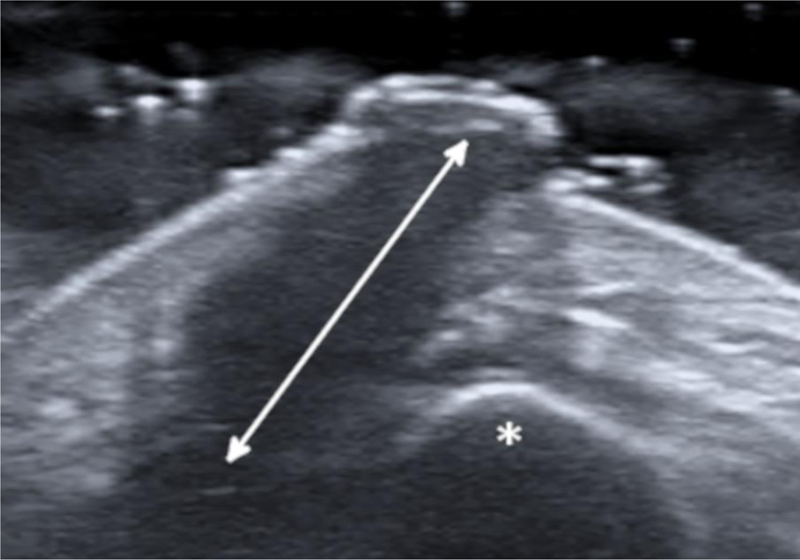
Ultrasonography examination reveals a wide hypoechoic band (arrow) extending through the subcutaneous tissue to the mandibular bone (asterisk).

**Figure 3:**
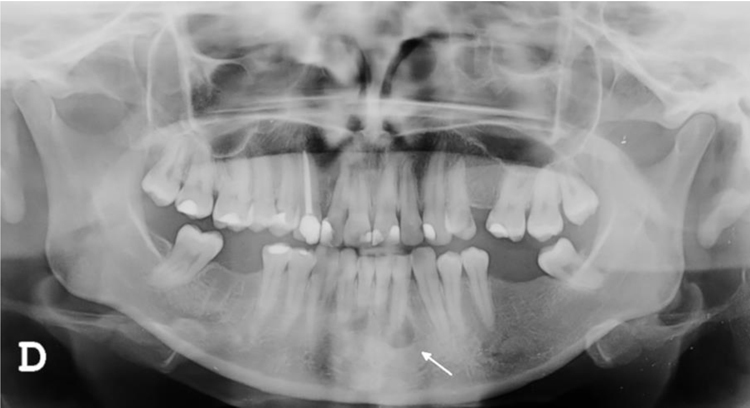
Panoramic radiograph showing radiolucency around the root of the mandibular left lateral incisor (arrow).

OCF is the result of an abnormal canalization originating from chronic periapical infection. Usually, bacterial infection from the dental pulp drains into the oral cavity. However, when the inflammatory process is severe and long-standing, it may destroy the alveolar bone, spread to the surrounding soft tissue, and eventually drain to the skin.^1^ Possible causes include chronic infection of the jaws, trauma, retained roots, and pulp disease. Deficient oral hygiene and surgical procedures with complications can also facilitate such cutaneous lesions. Skin manifestations can present as a dimpling, nodule, abscess, cyst, ulcer, or draining lesion in variable location depending on the affected teeth.^2^ The sinuses are more commonly associated with the infection of the mandibular teeth rather than the maxillary teeth, which will normally drain to the chin and jaw. Given these many different presentations and locations, OCF is often misdiagnosed, leading to inappropriate dermatological treatment and unnecessary antimicrobial therapy. Differential diagnosis includes subcutaneous mycosis, actinomycosis, osteomyelitis, neoplastic processes such as basal cell carcinoma or squamous cell carcinoma, epidermal cysts, and pyogenic and foreign body granulomas.^1^

The diagnosis of OCF is usually made based on panoramic radiograph and computed tomography that show the damage of the alveolar process. However, these tests require considerable time and money to perform and may have side effects. Skin ultrasonography is a noninvasive and emerging technique with proven usefulness in localized lesions.^3^ High frequency imaging not only provides robust qualitative and quantitative information on skin lesions but also on their surrounding tissues. Moreover, color Doppler ultrasonography gives information about its vascularization. Therefore, this technique has been found really useful in the diagnosis of cutaneous sinus tracts. Ultrasound image of OCF consists of a hypoechoic linear but slightly tortuous sinus tract that reaches the cortical bone, with an increased blood flow in the peripheral regions of the tract.^4^, ^5^ Most of these features characterize this lesion and easily distinguish it from other pathologies included in the clinical differential diagnosis. Regarding its treatment, antibiotic therapy brings an apparent healing, but if the source of infection is not eliminated, the sinus tract recurs in time. It is believed that high-frequency ultrasonography could also be useful in monitoring response to treatment by showing a decrease in the vascularization and a progressive reduction of the sinus tract. In addition, it could also allow the detection of early recurrences. In order to accomplish resolution, therapy has to focus towards the management of the dental infection, either with endodontic treatment or extraction.^2^ Surgical excision of the sinus tract is not usually necessary, as it heals spontaneously after the dental treatment.

Skin ultrasound is a safe and accessible tool for the diagnosis of OCF. The characteristic image of a hypoechoic sinus tract enhanced by color Doppler enables its diagnosis. Hence, neoplastic lesions are rapidly discarded, and many inappropriate treatments are avoided.

## Financial support

None declared.

## Authors’ contributions

Arcadi Altemir-Vidal: Approval of the final version of the manuscript; study conception and planning; preparation and writing of the manuscript; data collection, analysis, and interpretation; effective participation in research orientation; intellectual participation in propaedeutic and/or therapeutic conduct of studied cases; critical review of the literature; critical review of the manuscript.

Maribel Iglesias-Sancho: Approval of the final version of the manuscript; study conception and planning; preparation and writing of the manuscript; data collection, analysis, and interpretation; effective participation in research orientation; intellectual participation in propaedeutic and/or therapeutic conduct of studied cases; critical review of the literature; critical review of the manuscript.

Monica Quintana-Codina: Approval of the final version of the manuscript; study conception and planning; preparation and writing of the manuscript; data collection, analysis, and interpretation; effective participation in research orientation; intellectual participation in propaedeutic and/or therapeutic conduct of studied cases; critical review of the literature; critical review of the manuscript.

## Conflicts of interest

None declared.

## References

[1] Guevara-Gutiérrez E., Riera-Leal L., Gómez-Martínez M., Amezcua-Rosas G., Chávez-Vaca C.L., Tlacuilo-Parra A. (2015). Odontogenic cutaneous fistulas: clinical and epidemiologic characteristics of 75 cases. Int J Dermatol.

[2] Chhabra A., Chhabra N. (2018). Dental Infection Mimicking Dermatological Lesion: Three Case Reports of Cutaneous Fistulae and Sinus Tracts on Face. Indian Dermatol Online J.

[3] Wortsman X., Wortsman J. (2010). Clinical usefulness of variable-frequency ultrasound in localized lesions of the skin. J Am Acad Dermatol.

[4] Shobatake C., Miyagawa F., Fukumoto T., Hirai T., Kobayashi N., Asada H. (2014). Usefulness of ultrasonography for rapidly diagnosing cutaneous sinus tracts of dental origin. Eur J Dermatol.

[5] Garrido Colmenero C., Blasco Morente G., Latorre Fuentes J.M., Ruiz Villaverde R. (2015). Diagnostic Value of Color Doppler Ultrasound for Cutaneous Odontogenic Sinus Tract. Actas Dermosifiliogr.

